# Association of Polyps with Early-Onset Colorectal Cancer and Throughout Surveillance: Novel Clinical and Molecular Implications

**DOI:** 10.3390/cancers11121900

**Published:** 2019-11-29

**Authors:** José Perea García, Julia Arribas, Ángel Cañete, Juan Luis García, Edurne Álvaro, Sandra Tapial, Cristina Narváez, Alfredo Vivas, Lorena Brandáriz, Sergio Hernández-Villafranca, Daniel Rueda, Yolanda Rodríguez, Jessica Pérez-García, Susana Olmedillas-López, Damián García-Olmo, Giulia Martina Cavestro, Miguel Urioste, Ajay Goel, Rogelio González-Sarmiento

**Affiliations:** 1 Surgery Department, Fundación Jiménez Díaz University Hospital, 28040 Madrid, Spain; lorena.brandariz@gmail.com (L.B.); sergio.hernandezv@quironsalud.es (S.H.-V.); damian.garcia@uam.es (D.G.-O.); 2 Fundación Jiménez Díaz University Hospital Health Research Institute, 28040 Madrid, Spain; susana.olmedillas@fjd.es; 3 Gastroenterology Department, 12 de Octubre University Hospital, 28041 Madrid, Spain; jantiart@gmail.com (J.A.); canete_angel@hotmail.com (Á.C.); 4 Molecular Medicine Unit Department of Medicine, Biomedical Research Institute of Salamanca (IBSAL) and Institute of Molecular and Cellular Biology of Cancer (IBMCC), University of Salamanca-SACYL-CSIC, 37001 Salamanca, Spain; jlgarcia@usal.es (J.L.G.); jperezg84@hotmail.es (J.P.-G.); 5 Surgery Department, “Infanta Leonor” University Hospital, 28031 Madrid, Spain; eac.lomba@gmail.com; 6 Digestive Cancer Research Group, 12 de Octubre Research Institute, 28041 Madrid, Spain; stapialsantos@gmail.com (S.T.); druedafer.hdoc@gmail.com (D.R.); 7 Surgery Department, 12 de Octubre University Hospital, 28041 Madrid, Spain; cristhy_n@hotmail.com (C.N.); alfredovivas7@gmail.com (A.V.); 8 Molecular Biology Laboratory, 12 de Octubre University Hospital, 28041 Madrid, Spain; 9 Pathology Department, 12 de Octubre University Hospital, 28041 Madrid, Spain; yolandarodriguezgil@hotmail.com; 10 Gastroenterology and Gastrointestinal Endoscopy Unit, Division of Experimental Oncology, Vita-Salute San Raffaele University, IRCCS Ospedale San Raffaele Scientific Institute, 20132 Milan, Italy; cavestro.giuliamartina@hsr.it; 11 Human Genetics Group, Human Cancer Genetics Program, Spanish National Cancer Centre (CNIO), 28029 Madrid, Spain; 12 Centro de Investigación Biomédica en Red de Enfermedades Raras (CIBERER), Instituto de Salud Carlos III, 28029 Madrid, Spain; 13 Beckman Research Institute at City of Hope Comprehensive Cancer Center, 1218 S. Fifth Avenue, Monrovia, CA 91016, USA

**Keywords:** early-onset colorectal cancer, polyp development, prognosis, follow-up

## Abstract

Early-onset colorectal cancer (EOCRC) is an increasing and worrisome entity. The aim of this study was to analyze its association with polyps concerning prognosis and surveillance. EOCRC cases were compared regarding the presence or absence of associated polyps (clinical and molecular features), during a minimum of 7 years of follow-up. Of 119 cases, 56 (47%) did not develop polyps (NP group), while 63 (53%) did (P group). The NP group showed a predominant location of the CRC in the rectum (50%), of sporadic cases (54%), and diagnosis at advanced stages: Only *P53* and *SMARCB1* mutations were statistically linked to this group. The P group, including mainly early-diagnosed tumors, was linked with the most frequent and differential altered chromosomal regions in the array comparative genomic hybridization. The two most frequent groups according to the follow-up were the NP group (40%), and patients developing polyps in the first 5 years of follow-up (P < 5FU) (34%) (these last groups predominantly diagnosed at the earliest stage and with adenomatous polyps (45%)). EOCRC with polyps that developed during the entire follow-up (PDFU group) were mainly located in the right colon (53%), diagnosed in earlier stages, and 75% had a familial history of CRC. Patients developing polyps after the first 5 years (P > 5FU) showed a mucinous component (50%). Our results show that the absence or presence of polyps in EOCRC is an important prognostic factor with differential phenotypes. The development of polyps during surveillance shows that it is necessary to extend the follow-up time, also in those cases with microsatellite-stable EOCRC.

## 1. Introduction

Colorectal cancer (CRC) is the second leading cause of cancer-associated decease in the western world with respect to both incidence and mortality rate and is the most common tumor type in both sexes combined in Western countries [1]. The incidence of early-onset CRC (EOCRC) has been rising during the last decades, compared with onset in patients older than 49 years old; in this latter group, it has decreased, together with cancer-associated mortality, particularly in the USA [2]. According to the tendency observed at present, an increase of 90% and 124.2% is expected in the incidence rates of colon and rectal cancers, respectively, in the population aged 20 to 34 years old, whereas these rates will grow by 27.7% and 46.0%, respectively, in 35 to 39-year-old patients [3,4]. Moreover, the number of EOCRC-related deaths has not changed significantly between 1998 and 2012 (2.1/100,000 to 2.7/100,000) [5], despite the fact that an increase of 13% has been observed between 2000 and 2014 [6]. In Europe, until recently, data on CRC incidence among younger adults were lacking. The most recent studies show that, even though in most European countries the EOCRC incidence has risen, there is a wide spectrum of heterogeneity between countries [7,8]. During the most recent decade of available data, CRC incidence rates have uniquely increased in young adults in countries, such as Germany, the UK, Denmark, Slovenia, and Sweden, while conversely, CRC declined in young adults in only three countries (Italy, Austria and Lithuania); in Cyprus, the Netherlands, and Norway, inclines in the incidence in young adults were twice as rapid as those in older adults [7].

The decreasing incidence of CRC in the general population points to changing risk factor frequencies as well as the implementation of screening strategies [9]. Until recently, guidelines recommended that people under 50 years whose family’s history of CRC has been reported, patients with oncologic genetic syndromes, and patients suffering from inflammatory bowel disease for a long time should be subjected to CRC screening [10]. Estimations calculate that around 23% of EOCRC patients have a history of CRC in their families [9,11]. The increase in the incidence of EOCRC, together with the significant proportion of cases with sporadic characteristics within this subgroup of CRC have led the American Cancer Society to recommend starting regular screening by a stool-based test or a visual exam (endoscopy) at age 45 for people at average risk for CRC [12].

Nevertheless, there are established guidelines for the follow-up of patients surviving CRC. These usually include outpatient visits and clinical, hematological, radiological, and colonoscopic evaluation (the principal methods for the detection of metachronous CRC or polyps during follow-up). Postoperative follow-up plans have become very exhaustive, aiming at the early diagnosis of recurrence in asymptomatic patients, with the aspiration that this will help to identify a higher number of patients who are potential candidates for therapy with curative intent. In fact, there are several reviews and meta-analyses showing better survival with this approach [13,14,15].

While there are screening protocols for the general population and guidelines for the follow-up of CRC survivors, there are no established screening protocols for EOCRC patients or specific follow-up guidelines after CRC resection. There is still much left to do regarding the search for prognostic factors and guidelines for the postoperative follow-up of these patients, especially in cases in which there is no clear hereditary component. The aim of this study was firstly to analyze the relations between EOCRC, particularly “sporadic” EOCRC, and the development of polyps before/at diagnosis, and during follow-up, to identify possible clinical implications that may help us define future approaches, and also identify possible risk groups in the younger population with no defined family history of cancer. Secondly, this study aimed to evaluate the development of polyps during surveillance after EOCRC diagnosis and the need for a larger follow-up, independently from the familial cancer history.

## 2. Results

### 2.1. Overall Features

We studied 119 patients with EOCRC. In general terms, only 4 patients (3.4%) showed colon polyps prior to CRC development, while 33 cases (28%) developed polyps synchronous to the neoplasm. A total of 63 cases (53%) showed associated polyps at any time (before diagnosis and/or at diagnosis and/or during surveillance) (P group). Prior to the CRC diagnosis, a total of 55 cases (47%) had at least an endoscopic exam (20 from the NP group and 35 from the P group). All these cases were screened previously due to familial CRC history. Mixed polyps were the main polyp type observed (40%), followed by adenomatous polyps (32%): Only 18% and 2% were hyperplastic and serrated, respectively. Clinicopathological and familial features are shown in Table 1.

### 2.2. Comparative Analysis of EOCRC with and without Associated Polyps

#### 2.2.1. Clinicopathological and Familial Differences

**Figure 1:**
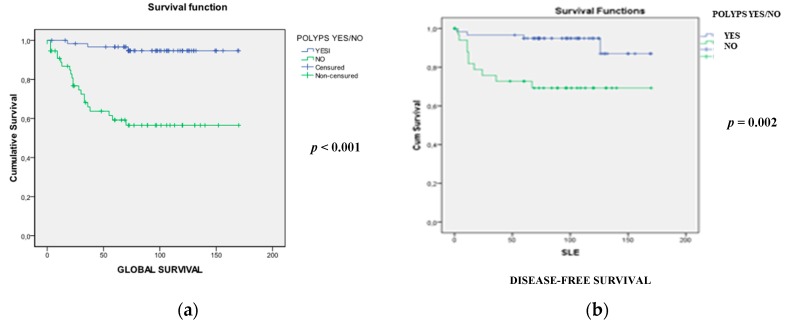
Comparative analysis of overall (**a**) and disease-free (**b**) survival between the polyp and no-polyp groups in early-onset colorectal cancer.

As shown in Table 1, 56 cases (47%) did not develop polyps (NP group). Tumors in the NP group were predominantly located in the rectum (50%) and infrequently in the right colon (16%), were mostly diagnosed in advanced stages (III and IV) (25% and 43%, respectively), and were mainly sporadic cases (54%). OS and DFS were significantly worse in the NP group (Table 1 and Figure 1a,b). On the other hand, in the polyps’ group (P), patients were mainly diagnosed in early stages and had a better prognosis. Regarding the familial cancer history, Lynch syndrome-neoplasm aggregation was substantially present (58%), and there were 30 (48%) CRC-aggregation cases.

#### 2.2.2. Molecular Differences

MSI and LS cases:

In total, 112 cases were analyzed for MSI, and positive cases were subsequently analyzed for germline Mismatch Repair (*MMR)* gene mutations or sporadic molecular features (*BRAF* and/or *MLH1* hypermethylation). Of the 112 cases, 19 (17%) showed MSI. Moreover, 11 out of 59 (19%) cases in the P group, and 6 out of 53 (11%) cases in the NP group were LS cases.

Other molecular analysis: NGS, chromosomal instability and CpG Island Methylator Phenotype (CIMP):

Excluding MSI cases, we selected two different subsets from both groups with correlative clinic-pathological features, in order to analyze further molecular characteristics: 19 cases from the P group and 21 from the NP group. Genes most frequently mutated in both groups together were *APC* (17/40, 42.5%), *P53* (15/40, 37.5%), *KRAS* (8/40, 20%), and *SMARCB1* (5/40, 12.5%). Considering the groups separately, only *P53* and *SMARCB1* cases were statistically different, linked to the NP group. *P53* was mutated in 10 out of 21 NP group cases (48%) vs. 5 out of 19 within the P group (26%). Regarding *SMARCB1*, four mutations were found in the NP group while only one case was observed in the P group.

The array comparative genomic hybridization (aCGH) showed that the most frequent gains in the P group were in 19p13 (84%), and in 7q22, 17q24-q25 and 19q13 (68% for each of them). The most frequent losses were in 14q11 (79%), 1p36 and 5q13 (74% each) and in 1p12-q21 (68%) (Table S1). On the other hand, the most frequent gains in the NP group were in 6p21, 17q11 and 20q11 (each of them 62%); the most frequent losses were in 14q11.1 (71%) and 9p12-p11 (69%) (Table S2). Statistically significant differentially altered chromosomal regions were all losses in 1p33-32.3 and 5p14.3 (47%), 3p22.2-21.31 (32%) and 1q32.1-32.2 and 5p15.2-15.1 (21%), all of which were related more frequently with the P group (Table 2). According to the genomic instability index, the P group showed more genomic instability, but the differences were not statistically significant.

Finally, we did not observe CIMP predominance in either group: Only three cases in each group showed CIMP-high (16% and 14% in the P group and the in the NP group, respectively).

### 2.3. Comparative Analysis of Patients with EOCRC with the Development of Polyps during the Subsequent Follow-Up

In total, 93 patients fulfilled the inclusion criteria for the second part of the study (development of polyps during follow-up after CRC diagnosis). The four different groups were as follows: NPDF group, with 37 cases (40%), in which patients did not develop polyps; the group of patients who developed polyps during the entire follow-up (PDFU), with 17 cases (18%); patients who developed polyps after the first 5 years of follow-up (P > 5FU), with 7 cases (8%); and finally, patients who developed polyps in the first 5 years of follow up (P < 5FU), with 32 cases (34%). Comparisons between these groups are shown in Table 3.

**Figure 2:**
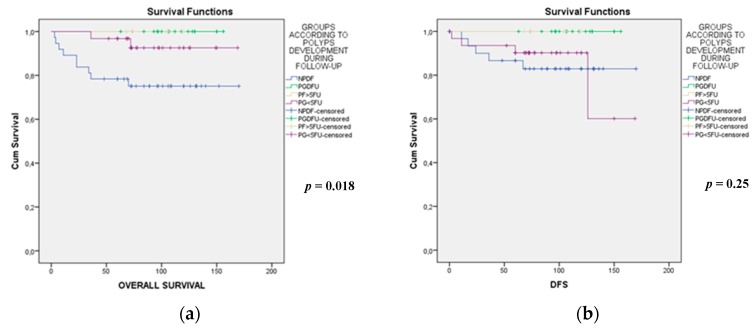
Comparative analysis of overall (**a**) and disease-free (**b**) survival between groups according to the development of polyps during follow-up. DFS: Disease-free survival. NPDF: No polyps during follow-up group. PGDU: polyp development during the entire follow-up group. PG > 5FU: Polyp development after 5 years of follow-up group; PG < 5FU: Polyp development before 5 years of follow-up group.

The NPDF group showed, as previously mentioned, a predominant tumor location in the left-side of the colon and the rectum (81%); the tumors were diagnosed in advanced stages (III and IV: 27% and 24%, respectively) and the patients showed a worse prognosis (Figure 2a,b) and had a larger sporadic component. Recurrence appeared only in the NPDF and P < 5FU groups (13% for both). Remarkably, CRCs in the PDFU group were mainly located in the right colon (53%) and were diagnosed in earlier stages; patients had the best prognosis (both OS and DFS) and had an important familial cancer history (CRC in 75%), and the type of polyps was mainly mixed (88%). The *P* > 5FU CRC group showed an important mucinous component (50%) and patients were diagnosed at intermediate stages (71% at stage II). Finally, the P < 5FU group showed the highest proportion of cases diagnosed at stage 0 (59%), did not show a mucinous component, and the type of polyps were more frequently adenomatous (45%). Familial cancer histories in the latter two groups were equivalent and were at an intermediate level between the NPDF and PDFU groups. Only two cases developed adenomatous polyps with a villous component, both in the first colonoscopy, one of them corresponding to the group of PDFU while the other corresponded to the group of P < 5FU.

## 3. Discussion

EOCRC has a repercussion of unquestionable relevance on the population and its incidence is increasing. The proportion of hereditary forms is estimated to be less than 20%, i.e., most cases are sporadic [16]. It is therefore important to obtain as much information as possible on the behavior of EOCRC. Two important and intimately related factors are prognosis and follow-up. During our analysis of the development of polyps within EOCRC, we found that this provided important information for the prognosis of patients. Many factors have been studied in relation with CRC prognosis and this also applies, in part specifically, to EOCRC. The issue of prognosis of EOCRC itself has been a subject of debate for some time: Several studies suggested that this type of CRC was associated with poorer prognosis, but others suggested more variability in outcomes [4,17,18,19]. The association or not of polyps with EOCRC appears important in our study in relation to prognosis. The better prognosis of the P group was not related to a previous comprehensive screening for hereditary CRC (regarding LS cases in both groups) or the presence of polyps synchronous with CRC. Nonetheless, 47% had at least one colonoscopy because of a familial CRC history prior to CRC diagnosis (20 from the NP group and 35 from the P group) and the proportions of earlier stages at diagnosis (I–II) were slightly, but not remarkably, in favor of the P group. On the other hand, in the NP group, advanced stage at diagnosis is a factor that affects the prognosis negatively. The higher frequency of rectal tumors should also be noted, as the increase in the incidence of EOCRC is mainly due to an increase at this location [3,4,11,20].

Given the importance of identifying EOCRC cases with or without associated polyps, especially those developing after the tumor, it is mandatory that markers are found that can differentiate between both groups. The NP CRC group may be a group with a more aggressive biological behavior, since *P53* mutations were significantly increased in this group and mutations in this gene have been associated with poor prognosis in CRC [21,22,23]. Although we are aware that a weakness of our study lies in the small subsets of the P and NP groups used for molecular analysis, our results showed interesting altered chromosomal regions that can serve as starting points for specific studies aimed at determining whether they can be used for the identification of suitable markers. For example, in the P group, chromosomal region 19p13 is the most frequently gained region: A gene encoded by this region is *DDA1*, which may be involved in the activation of nuclear factor kappaB (NFκB) and tumor progression [24]. Interestingly, this region has also been shown to be amplified in CRC [25]. The most frequently lost region in the P group is 14q11. This is a CNV region that encompasses the chromatin modifier *CHD8*, which has been significantly associated with sporadic CRC risk [26]. Regarding the NP group, in 6p21 (one of the most frequently gained regions), *VEGFA* is located, which encodes a growth factor from the PDGF/VEGF family, responsible for the induction of vascular endothelial cell migration and proliferation, thus playing a pivotal role in angiogenesis, both in physiological and pathological conditions [27]. In one of the most frequently lost regions, in 9p11, microRNA-1299 is encoded, a negative CRC regulator of *STAT3* that is essential for cancer progression to advanced malignancy [28]. Finally, regarding the differentially altered chromosomal regions, the gene encoding cadherin-12 is located in 5p14.3; this protein enhances proliferation of CRC cells and increases progression by promoting epithelial-mesenchymal transition [29].

Another important point is the one related to EOCRC surveillance. According to our results, the PDFU group appeared to present a hereditary form of CRC and/or polyposis, not only because of the continuous tendency to develop polyps over time but also because of the existence of a familial cancer history, mainly related to CRC. Genetic counseling and testing with a multigene panel could be considered especially for this group of patients (but also for the rest of the cases), as well as approaches to find new susceptibility genes within this subset of cases. Moreover, it is important to reconsider the surveillance of EOCRC patients, since a long-term follow-up with endoscopic surveillance is essential. This is necessary not only to detect patients who may develop polyps at any given time but, more importantly, patients who develop polyps only after the first five years of follow-up.

## 4. Materials and Methods

### 4.1. Families, Samples and Data Collection

We selected patients diagnosed with CRC at an age of 45 years old or younger from January 2002 to December 2011 from a single hospital institution. We selected 45 years as the cut-off age because screening starts to begin at that age, at least in the US [12], and therefore people younger are those left without it, and thus, at a higher risk. They were considered the index case of each family. As a first step in the study, we excluded those cases with a diagnosis of inflammatory bowel disease, familial adenomatous polyposis (FAP), MYH-associated polyposis, and other types of inheritable polyposis (hyperplastic or serrated).

Written informed consent was obtained from all patients or a first-degree relative in the event of index case decease. The ethical board committee of our institution reviewed and approved this study (PI16-01650). From each patient, paraffin-embedded colorectal tumor tissue was collected in addition to recording the full family history of cancer over three generations. A complete review of pathological and clinical reports was performed in order to verify cancer diagnosis.

Clinical, pathological and personal data were collected including the following information: Gender, age of onset, tumor location (right colon, left colon or rectum), TNM tumor stage, cell differentiation grade (low, medium or high), presence of “signet ring” cells, production of mucin, and diagnosis of synchronous or metachronous CRC in the index case. Family history of cancer was analyzed classifying families into four groups: (a) Families with aggregation (at least one family member) of Lynch syndrome (LS)-related neoplasms, including CRC [30]; (b) families with only specific aggregation of CRC; (c) families uniquely with aggregation of Lynch syndrome-unrelated neoplasms; and d) cases not presenting familial cancer history, who were considered sporadic cases.

Each case had a minimum follow-up period of 5 years from primary diagnosis with a complete report of recurrence, disease-free survival (DFS) and overall survival (OS) (in months), death, and cancer-related death. The periodicity of colonoscopy during follow-up was every 3 years, in addition to the corresponding colonoscopy of the first year in cases in which CRC surgery was performed at diagnosis, except when a colonic polyp was diagnosed; in the latter case, colonoscopy was carried out the following year.

Associated-polyp cases were defined in at least one of the following settings: Finding before diagnosis of CRC, at diagnosis of CRC (synchronous), or during follow-up. Therefore, two groups were initially defined: Cases with (polyps’ group; P) or without associated polyps (no polyps’ group; NP). We classified polyp-group cases according to the anatomopathological type of the polyps, as follows: Cases associated with only adenomatous polyps; only hyperplastic, only serrated, or mixed (cases associated with different types during natural history: Adenomatous, hyperplastic, and serrated). We also defined cases developing polyps with a villous component. In the second part of the study, we defined different categories according to the association with polyp development during different moments of surveillance after CRC diagnosis, considering only cases with at least 7 years of follow-up, and discarding cases with LS. We defined four categories: No polyps during follow-up (NPDF); polyp development during the entire follow up (PDFU); polyp development after 5 years of follow-up (P > 5FU); and polyp development before 5 years of follow up (P < 5FU).

### 4.2. Analysis of Microsatellite Instability (MSI) and Mutations in Mismatch Repair (MMR) Genes

Tumor and healthy tissues selection of the index cases, tumoral cells proportion, and DNA extraction have been described before [31].

First, we classified tumors according to their microsatellite status, and MSI cases were further analyzed to see if they were linked to LS due to a germline mutation in an *MMR* gene or if they were sporadic cases. The Bethesda panel was used for MSI analysis, as previously described [31]. When two or more of the five markers showed instability, tumors were classified as MSI-H (high frequency of MSI), whereas the rest of the tumors were considered as microsatellite stable (MSS).

The sporadic nature of MSI tumors was initially confirmed by the analysis of the *BRAF* V600E mutation and hypermethylation of the *MLH1* gene promoter. In case of a negative result, the presence of germline mutations in *MLH1*, *MSH2*, *MSH6* and *PMS2 (*DNA mismatch repair genes) was analyzed, as previously described [31].

### 4.3. Other Molecular Analyses

We selected correlative samples from the P and NP groups (with and without associated polyps), after ruling out cases with LS and sporadic MSI cases. We analyzed the mutational profiles, chromosomal instability, and possible differentially altered chromosomal regions, and CpG island methylator phenotype (CIMP).

#### 4.3.1. Mutational Status Analysis by Next Generation Sequencing

The methods used have already been described previously for other samples (e.g., synchronous CRCs) [32]. In short, for all samples, the following steps were carried out: Ion torrent PGM library preparation; emulsion PCR; sequencing on the Ion torrent PGM platform; and Bioinformatics processing and data analysis. The genes included are listed in Table S3.

#### 4.3.2. Chromosomal Instability

Oligonucleotide microarrays (Roche NimbleGen, Inc., Reykjavik, Iceland) were used to perform an aCGH, as previously described [33], aimed at the identification of copy number alterations (CNAs) for both polyp- and non-polyp-associated subgroups. CNAs larger than 0.5 MB were considered. The definition of genomic instability degrees has also been described before [30]. We included the CNAs in the Gene Expression Omnibus (GEO) (GSE108220) (http://www.ncbi.nlm.nih.gov/geo/).

#### 4.3.3. CpG Island Methylator Phenotype

We established three categories for patients regarding the methylation status of the promoter regions of *CACNA1G*, *CDKN2A*, *CRABP1*, *IGF2*, *MLH1*, *NEUROG1*, *RUNX3* and *SOCS1* genes in their tumors: CIMP-high when tumors presented ≥ 5/8 methylated promoters, CIMP-low if this number ranged from 2/8 to 4/8, and CIMP-0 in the case of exhibiting 0/8 to 1/8 methylated promoters. 

The methodology used to evaluate CIMP has been previously described [31].

### 4.4. Statistical Analyses

Categorical variables (expressed as number of cases and percentage) were compared using Pearson’s Chi Square (χ^2^) test. Student’s t-Test was used for independent samples and the Mann–Whitney U test was used for continuous variables (expressed as mean values plus/minus standard deviation (SD)). Analysis of variance (ANOVA) was used to compare more than two groups following normal distributions and the Kruskal–Wallis test for those following nonparametric distributions. The relationship between associated polyps´ groups and OS and DFS was analyzed by the Kaplan–Meier method (log-rank test). SPSS version 23.0 (IBM) was used for statistical analyses. A *p*-Value < 0.05 was established to consider differences as statistically significant.

## 5. Conclusions

Our results show that the absence or presence of polyps associated with EOCRC is an important prognostic factor, and that respective CRCs have different clinical and molecular phenotypes. Differences in the timing of the development of polyps during surveillance make it necessary to extend follow-up over time. Molecular markers must be identified that enable us to predict at the time of CRC diagnosis with a reasonable degree of accuracy if and when a patient will develop polyps. This should be important not only for prognosis and follow-up but also for the primary treatment of EOCRC. Thus, the need to identify risk groups within the younger population with no family history of cancer becomes even more essential in the specific situation we showed with this study: EOCRC potentially without association with other polyps.

## Figures and Tables

**Table 1:** Clinical and pathological characteristics of the whole cohort of early-onset CRC patients, including the family history of cancer and comparing groups with and without associated polyps.

	Global *n* (%)	No polyps *n* (%)	Polyps *n* (%)	*p* (χ^2^)
Patients	119 (100)	56 (47)	63 (53)	−
Mean age of onset (±SD) ^1^ (years)	40.7 (±4.4)	41.0 (±4.4)	40.6 (±4.1)	NS
Sex:				
Male	67 (56)	37 (55)	30 (45)	NS
Female	52 (44)	26 (50)	26 (50)	
Colon location:				0.064
Right	29 (25)	9 (16)	20 (32)	
Left	42 (35)	19 (34)	23 (36)	
Rectum	48 (40)	28 (50)	20 (32)	
Mucin production ^2^	21/79 (26)	16/48 (33)	5/31 (16)	NS
“Signet ring” cells ^2^	3/98 (3)	2/51 (4)	1/47 (2)	NS
Stage at diagnosis:				<0.001
I	47 (39)	9 (16)	38 (60)	
II	25 (21)	9 (16)	16 (25)	
III	18 (15)	14 (25)	4 (6)	
IV	29 (24)	24 (43)	5 (8)	
Overall survival (±SD) ^1^ (months)	76.7 (±45.6)	58.8 (±47.1)	94.5 (±36.6)	0.012
Disease-free survival (±SD) ^1^ (months)	68.1(±51.9)	45.3 (±52.2)	90.6 (±40.7)	<0.001
Synchronous CRCs	5/119 (4)	1/56 (2)	4/63 (6)	NS
Family history of cancer				
Aggregation for Lynch-related neoplasm	56 (48)	20 (37)	36 (58)	0.027
Aggregation for CRC	43 (37)	13 (24)	30 (48)	0.007
Aggregation for Lynch-unrelated neoplasm	10 (13)	13 (24)	23 (37)	NS
Sporadic cases	33 (41)	30 (54)	16 (26)	0.002

^1^ Statistical analysis was performed using the Student’s *t* Test. ^2^ Percentages shown were calculated from different numbers of total cases: “in situ” carcinomas with severe dysplasia were excluded because there was no possibility to study any other feature. Cases in which a single biopsy was taken (stage D) were also excluded. SD: Standard Deviation. NS: Not significant. CRC: Colorectal Cancer.

**Table 2:** Chromosomal regions differentially altered in the P and NP groups (statistically significant).

Chr	Start	End	Region	Region	Size (Mb)	P Group % Gains	% Losses	NP Group % Gains	% Losses
chr 1	49547237	50769506	*p*33	p32.3	1,222269	5,263157895	47,36842105	4,761904762	14,28571429
chr 1	50819987	52195285	*p*32.3	p32.3	1,375298	26,31578947	26,31578947	23,80952381	0
chr 1	204672629	208121292	*q*32.1	q32.2	3,448663	0	21,05263158	9,523809524	0
chr 3	38942563	46420768	*p*22.2	p21.31	7,478205	5,263157895	31,57894737	19,04761905	5,263157895
chr 5	14473935	16465030	*p*15.2	p15.1	1,991095	15,78947368	21,05263158	14,28571429	0
chr 5	20785106	21941459	*p*14.3	p14.3	1,156353	5,263157895	47,36842105	14,28571429	14,28571429

P: Polyps. NP: No Polyps. Chr: Chromosome.

**Table 3:** Clinical, pathological, and familial features of the groups of patients without associated polyps or with different timings of polyp development during follow-up in early-onset CRC.

	No Polyps During Follow-Up (NPDF) *n* (%)	Polyp Development During Follow-Up (PDFU) *n* (%)	Polyp Development after 5 Years of Follow-Up (P > 5FU) *n* (%)	Polyp Development before 5 Years of Follow-Up (P < 5FU) *n* (%)	*p* (χ^2^)
Patients	37	17	7	32	−
Mean age of onset (±SD) ^1^ (years)	40.8 (±4.4)	39.2 (±4.4)	40.4 (±5)	41.5 (±3.6)	NS
Sex:					
Male	20 (54)	8 (47)	5 (71)	21 (66)	
Female	17 (46)	9 (53)	2 (29)	11 (34)	NS
Colon location:					NS
Right	7 (19)	9 (53)	2 (29)	8 (25)	
Left	13 (35)	3 (18)	4 (57)	13 (41)	
Rectum	17 (46)	5 (29)	1 (14)	11 (34)	
Type of surgery:					
Curative	30 (81)	17 (100)	7 (100)	31 (97)	0.037
Palliative	7 (19)	0 (0)	0 (0)	1 (3)	
Mucin production ^2^	9/29 (31)	3/8 (38)	3/6 (50)	0/14 (0)	0.055
“Signet ring” cells	2/31 (7)	0/11 (0)	0/7 (0)	1/26 (4)	NS
Stage at diagnosis:					
I	12 (33)	10 (59)	2 (28)	21 (65)	
II	6 (16)	4 (24)	5 (71)	8 (25)	
III	10 (27)	2 (12)	0 (0)	2 (6)	0.003
IV	9 (24)	1 (6)	0 (0)	1 (3)	
Overall survival (±SD) ^1^ (months)	84 (±45)	114 (±25)	88 (±21)	88 (±34)	0.04
Disease-free survival (±SD) ^1^ (months)	74 (±51)	114 (±25)	88 (±21)	81 (±38)	0.012
Synchronous CRCs	1 (3)	2 (12)	0 (0)	1 (3)	NS
Family history of cancer					
Aggregation for Lynch-related neoplasm	16/37 (43)	12/17 (71)	4/7 (57)	16/32 (50)	NS
Aggregation for CRC neoplasm	12/37 (32)	12/17 (71)	3/7 (43)	12/32 (38)	0.032
Aggregation for Lynch-unrelated neoplasm	11/37 (30)	8/17 (47)	3/7 (32)	7/32 (22)	NS
Sporadic cases	18/37 (49)	2/17 (12)	2/7 (29)	11/32 (34)	0.069

^1^ Statistical analysis was performed using the Student’s *t* Test. ^2^ Percentages shown were calculated from different numbers of total cases: “in situ” carcinomas with severe dysplasia were excluded because there was no possibility to study any other feature. Cases in which a single biopsy was taken (stage D) were also excluded. SD: Standard Deviation. NS: Not significant. CRC: Colorectal Cancer.

## Supplementary Materials

The following are available online at https://www.mdpi.com/2072-6694/11/12/1900/s1. Table S1: Segmental chromosomal alterations within Polyps group, Table S2: Segmental chromosomal alterations within No Polyps group, Table S3: List of genes in which mutational status was analyzed by Next Generation Sequencing.
