# Supplementary material for: Association of Polyps with Early-Onset Colorectal Cancer and Throughout Surveillance: Novel Clinical and Molecular Implications
Source: Cancers (Basel). 2019 Nov 29;11(12):1900. doi: 10.3390/cancers11121900 (PMC6966640; doi:10.3390/cancers11121900)
Supplement: Supplementary file 1 [file cancers-11-01900-s001.pdf]

Supplementary Materials

Association of Polyps with Early-Onset Colorectal Cancer and Throughout Surveillance: Novel Clinical and Molecular Implications

José Perea, Julia Arribas, Ángel Cañete, Juan Luis García, Edurne Álvaro, Sandra Tapial, Cristina Narváez, Alfredo Vivas, Lorena Brandáriz, Sergio Hernández-Villafranca, Daniel Rueda, Yolanda Rodríguez, Jessica Pérez-García, Susana Olmedillas-López, Damián García-Olmo, Giulia Martina Cavestro, Miguel Urioste, Ajay Goel and Rogelio González-Sarmiento

Table S1. Segmental chromosomal alterations within the polyps group.

	Chromosome	Start	End	Cytoband		Size (Mb)	Cases	%
**Gains**	chr19	234741	8613258	p13.3	p13.2	8,378517	16	84
	chr19	9051563	19649923	p13.2	p13.11	10,59836	16	84
	chr19	8617097	9047690	p13.2	p13.2	0,430593	15	79
	chr19	43159132	55855553	q13.2	q13.42	12,696421	13	68
	chr19	63302460	63782948	q13.43	q13.43	0,480488	13	68
	chr7	99778118	101726777	q22.1	q22.1	1,948659	13	68
	chr17	70404330	71849942	q24.3	q25.1	1,445612	13	68
	chr17	76383844	78637182	q25.3	q25.3	2,253338	13	68
	chr19	19919560	20016774	p13.11	p12	0,097214	12	63
	chr19	40122442	43157699	q13.2	q13.2	3,035257	12	63
	chr19	55900538	60934428	q13.42	q13.43	5,03389	12	63
	chr19	63091436	63300085	q13.43	q13.43	0,208649	12	63
	chr20	29836272	36137344	q11.21	q11.23	6,301072	12	63
	chr20	41246969	49984597	q12	q13.2	8,737628	12	63
	chr22	16001612	16565146	q11.1	q11.1	0,563534	12	63
	chr7	504641	2759707	p22.3	p22.3	2,255066	12	63
	chr7	72555116	73714406	q11.23	q11.23	1,15929	12	63
	chr7	97538236	99774487	q21.3	q22.1	2,236251	12	63
	chr9	125876028	137212050	q33.3	q34.2	11,336022	12	63
	chr9	137857742	140089577	q34.3	q34.3	2,231835	12	63
	chr12	120462512	122864160	q24.23	q24.31	2,401648	12	63
	chr13	109959057	110297087	q33.3	q33.3	0,33803	12	63
	chr14	101794453	103269593	q32.31	q32.32	1,47514	12	63
	chr16	65226026	68494049	q21	q22.1	3,268023	12	63
	chr17	43084532	46333076	q21.31	q21.32	3,248544	12	63
	chr17	71912176	76374581	q25.1	q25.3	4,462405	12	63
**Losses**	chr14	18407780	19124665	q11.1	q11.2	0,716885	15	79
	chr1	37196	560407	p36.33	p36.33	0,523211	14	74
	chr5	69647605	70690846	q13.2	q13.2	1,043241	14	74
	chr1	580411	750291	p36.33	p36.33	0,16988	13	68
	chr1	120374173	143895571	p12	q21.1	23,521398	13	68
	chr5	69311609	69454785	q13.2	q13.2	0,143176	13	68
	chr9	41895789	46894470	p12	p11.2	4,998681	13	68
	chr9	67374725	69975267	q13	q21.11	2,600542	13	68
	chr10	48566509	48626138	q11.22	q11.22	0,059629	13	68
	chr21	13469523	13788440	q11.1	q11.1	0,318917	13	68
	chr1	144500777	144849473	q21.1	q21.1	0,348696	12	63
	chr1	146145351	147875429	q21.1	q21.2	1,730078	12	63
	chr5	69066506	69304673	q13.2	q13.2	0,238167	12	63
	chr7	76330593	76409573	q11.23	q11.23	0,07898	12	63
	chr7	143590133	143704676	q35	q35	0,114543	12	63
	chr9	47091550	67271885	p11.2	q13	20,180335	12	63
	chr10	46090375	46793103	q11.21	q11.22	0,702728	12	63

chr10	48376006	48562926	q11.22	q11.22	0,18692	12	63
chr10	48652320	49059190	q11.22	q11.22	0,40687	12	63
chr10	50744325	51006574	q11.23	q11.23	0,262249	12	63
chr10	89149827	89240951	q23.2	q23.2	0,091124	12	63
chr11	49049762	54821358	p11.12	q11	5,771596	12	63
chr11	88208207	89562713	q14.2	q14.3	1,354506	12	63
chr7	76422759	76619696	q11.23	q11.23	0,196937	11	58
chr9	41761730	41880647	p12	p12	0,118917	11	58
chr10	46059139	46089677	q11.21	q11.21	0,030538	11	58
chr10	51029772	51630099	q11.23	q11.23	0,600327	11	58
chr11	90444170	92131843	q14.3	q14.3	1,687673	11	58
chr15	18422770	20269549	p11.1	q11.1	1,846779	11	58
chr15	26152619	26864659	q12	q12	0,71204	11	58
chr16	69719425	69756045	q22.1	q22.1	0,03662	11	58
chr18	60263265	63245836	q21.33	q22.1	2,982571	11	58

Table S2. Segmental chromosomal alterations within the no-polyps group.

	Chr	Start	End	Cytoband		Size (Mb)	Cases	%
**Gains**	chr6	36077944	37539613	p21.31	p21.2	1.461669	13	62
	chr12	52184041	52193801	q13.13	q13.13	0.00976	13	62
	chr17	31348922	31969990	q11.2	q12	0.621068	13	62
	chr17	32238688	40823133	q12	q21.2	8.584445	13	62
	chr17	41056693	41655311	q21.31	q21.31	0.598618	13	62
	chr17	42230266	46335205	q21.31	q21.32	4.104939	13	62
	chr20	30331751	36041548	q11.21	q11.23	5.709797	13	62
	chr3	47089939	53031034	p21.31	p21.1	5.941095	12	58
	chr6	37548788	37683149	p21.2	p21.2	0.134361	12	58
	chr7	520082	1314300	p22.3	p22.3	0.794218	12	58
	chr11	64794916	64846593	q13.1	q13.1	0.051677	12	58
	chr12	51688436	52160236	q13.13	q13.13	0.4718	12	58
	chr12	52213748	53115661	q13.13	q13.13	0.901913	12	58
	chr12	54257456	55326755	q13.13	q13.2	1.069299	12	58
	chr17	31263448	31332087	q11.2	q11.2	0.068639	12	58
	chr17	32067491	32197322	q12	q12	0.129831	12	58
	chr17	40878942	41034225	q21.2	q21.31	0.155283	12	58
	chr17	41709705	41756011	q21.31	q21.31	0.046306	12	58
	chr17	52636376	52637426	q22	q22	0.00105	12	58
	chr17	69674564	78637182	q24.3	q25.3	8.962618	12	58
	chr19	234741	18226193	p13.3	p13.11	17.991452	12	58
	chr19	43208284	61099043	q13.2	q13.43	17.890759	12	58
	chr19	63177255	63729064	q13.43	q13.43	0.551809	12	58
	chr20	29306527	30280776	q11.1	q11.21	0.974249	12	58
	chr20	36060190	36249500	q11.23	q11.23	0.18931	12	58
	chr3	53089691	53142494	p21.1	p21.1	0.052803	11	54
	chr6	27321652	29194050	p22.1	p22.1	1.872398	11	54
	chr6	29709434	36048790	p22.1	p21.31	6.339356	11	54
	chr6	37684303	37907432	p21.2	p21.2	0.223129	11	54
	chr7	1387418	2966867	p22.3	p22.2	1.579449	11	54
	chr7	72591869	73733199	q11.23	q11.23	1.14133	11	54
	chr7	101106538	101332040	q22.1	q22.1	0.225502	11	54
	chr11	64713432	66898603	q13.1	q13.2	2.185171	11	54

	chr12	47371209	50279525	q13.11	q13.12	2.908316	11	54
	chr12	51579531	51622331	q13.13	q13.13	0.0428	11	54
	chr12	53119351	54176913	q13.13	q13.13	1.057562	11	54
	chr16	95546	3303856	p13.3	p13.3	3.20831	11	54
	chr17	31198895	31249514	q11.2	q11.2	0.050619	11	54
	chr17	32042027	32049105	q12	q12	0.007078	11	54
	chr17	41788252	42121317	q21.31	q21.31	0.333065	11	54
	chr17	46340040	46694286	q21.32	q21.32	0.354246	11	54
	chr17	52409474	52598290	q22	q22	0.188816	11	54
	chr17	52643187	61183245	q22	q23.3	8.540058	11	54
	chr17	61642331	63595203	q23.3	q24.1	1.952872	11	54
	chr17	69494756	69614543	q24.3	q24.3	0.119787	11	54
	chr19	18309191	19649923	p13.11	p13.11	1.340732	11	54
	chr19	40279210	43187150	q13.2	q13.2	2.90794	11	54
	chr19	62037657	63146643	q13.43	q13.43	1.108986	11	54
	chr19	63734841	63782948	q13.43	q13.43	0.048107	11	54
	chr20	25859325	26123443	p11.1	q11.1	0.264118	11	54
	chr20	61785859	62253823	q13.33	q13.33	0.467964	11	54
	chr22	30011302	30379359	q12.2	q12.2	0.368057	11	54
	chr22	38769797	41741808	q13.1	q13.2	2.972011	11	54
	chr22	48110394	49565997	q13.31	p22.33	1.455603	11	54
**Losses**	chr9	43653297	43817861	p11.2	p11.2	0.164564	15	71
	chr14	18407780	18943566	q11.1	q11.1	0.535786	15	71
	chr9	41895789	43613931	p12	p11.2	1.718142	14	67
	chr9	43858133	45095245	p11.2	p11.2	1.237112	14	67
	chr9	45284971	46893274	p11.2	p11.2	1.608303	14	67
	chr9	40504545	40765751	p13.1	p13.1	0.261206	14	67
	chr14	18990753	19124665	q11.1	q11.2	0.133912	14	67
	chr9	45107410	45189052	p11.2	p11.2	0.081642	13	62
	chr9	40774962	41880647	p13.1	p12	1.105685	13	62
	chr9	46894411	70059829	p11.2	q21.11	23.165418	13	62
	chr1	37196	547708	p36.33	p36.33	0.510512	13	62
	chr9	39461410	40064000	p13.1	p13.1	0.60259	13	62
	chr9	39054171	39453144	p13.1	p13.1	0.398973	12	57
	chr9	40320143	40415828	p13.1	p13.1	0.095685	11	53
	chr9	40095541	40165112	p13.1	p13.1	0.069571	11	52
	chr1	120556609	143895571	p12	q21.1	23.338962	11	52
	chr10	48376006	48562926	q11.22	q11.22	0.18692	11	52
	chr8	6864661	7217247	p23.1	p23.1	0.352586	11	52
	chr10	45613178	47045610	q11.21	q11.22	1.432432	11	52

Table S3. List of genes in which mutational status was analyzed by next generation sequencing.

ALB1	***JAK2***
AKT1	*JAK3*
ALK	*IDH2*
APC	*KDR*

ATM	*KIT*
BRAF	*KRAS*
CDH1	*MET*
CDKN2A	*MLH1*
CSF1R	*MPL*
CTNNB1	*NOTCH1*
EGFR	*NPM1*
ERBB2	*NRAS*
ERBB4	*PDGFRA*
EZH2	*PIK3CA*
FBXW7	*PTEN*
FGFR1	*PTPN11*
FGFR2	*RB1*
FGFR3	*RET*
FLT3	*SMAD4*
GNA11	*SMARCB1*
GNAS	*SMO*
GNAQ	*SRC*
HNF1A	*STK11*
HRAS	*TP53*
IDH1	*VHL*

© 2019 by the authors. Licensee MDPI, Basel, Switzerland. This article is an open access article distributed under the terms and conditions of the Creative Commons Attribution (CC BY) license (http://creativecommons.org/licenses/by/4.0/).
